# Dataset on the phagocytic and oxidative capacity of monocytes and granulocytes in the combined therapy of *S.aureus* infection in mice

**DOI:** 10.1016/j.dib.2023.109328

**Published:** 2023-06-17

**Authors:** Tamara A. Bukeyeva, Sabina G. Murzageldinova, Aray A. Serikbay, Arkadiy V. Krasnoshtanov, Ilya S. Korotetskiy

**Affiliations:** JSC Scientific Center for Anti-Infectious Drugs, Almaty, Kazakhstan

**Keywords:** Phagocytosis, Oxidative activity, Immune cells, Bacterial infections, Coordination compound, Cefazolin

## Abstract

The aim of this study was to evaluate phagocytic and oxidative activities of monocytes and granulocytes during combined therapy of mice infected by drug-resistant *Staphylococcus aureus* SCAID OTT1-2022. The treatment of the infected mice was conducted by using an iodine-containing coordination compound CC-195, antibiotic cefazolin and by a combined therapy with CC-195 and cefazolin. The PHAGOTEST and BURSTTEST kits (BD Biosciences, USA) were used to determine the phagocytic and oxidative activities. The samples were analyzed on a FACSCalibur flow cytometer (BD Biosciences, United States). It was found that the different treatment regiments applied on the infected animals caused a statistically significant differentiation in numbers and activity of monocytes and granulocytes in comparison to the negative and positive control animals (intact and infected but untreated mice, respectively).

**Specifications Table**

**Table:** 

Subject	Immunology
Specific subject area	Immunology of infectious diseases
Type of data	Tables, Imaging flow cytometry data
How the data were acquired	Collection: Samples were collected from mice treated by cefazolin and the coordination compound CC-195. FACSCalibur flow cytometer (BD Biosciences, United States) was used to generate raw data. Software: The CellQuest Pro BD software was used to process the raw data.
Data format	Analyzed data, *raw data.*
Description of data collection	Blood was collected into test tubes containing heparin as an anticoagulant. The PHAGOTEST and BURSTTEST kits (BD Biosciences, USA) were used to determine phagocytic and oxidative activities. Significance of the differences between the experimental data was assessed using GraphPad Prism version 6.00 for Windows (GraphPad Software, La Jolla California USA), using Two-way ANOVA.
Data source location	• Institution: Scientific Center for Anti-Infectious Drugs • City/Region: Almaty • Country: Kazakhstan • Latitude and longitude: 43°21′ N and 76°91′ E
Data acsessibility	Repository name: Mendeley: DataData identification number: http://dx.doi.org/10.17632/99hr4fn894.2

## Value of the Data

• These data will help in finding new ways to combat drug resistance of pathogens by developing more effective approaches of combinatorial immunotherapy of infectious diseases.
• Understanding mechanisms of function of immune cells during a response to bacterial infections will help to discover new treatment approaches to improve the immune system's capability to combat the infection.
• This may help identifying new targets for antibiotic development, which will more effectively target bacterial pathogens.
• Contribution to the development of new, more effective therapies against *S. aureus* infection.

## 1 Objective

Antibiotic therapy has been a cornerstone of the modern medicine for treating bacterial diseases for many years. However, the emergence of antibiotic-resistant bacteria has highlighted the need in better in-depth understanding on how the antibiotics function. A major achievement of this study is the demonstration of an impact of antibiotics on the phagocytic and oxidative activities of monocytes and granulocytes, which are critical for the immune response of the organism to bacterial infections.

Data was obtained during as an implementation of the ID #BR09458960 grant provided to improve our understanding of the effect of combined therapy on the host immune system challenged by an infection caused by a multidrug resistant *S. aureus* strain. This data will be useful to better understand the effect of the combined therapy by antibiotics and iodine-containing coordination compound on bacterial infections through activation of the phagocytic and oxidative activities of monocytes and granulocytes.

## 2 Data Description

The treatment of a patient with antimicrobial drugs is associated with a complex immunological reaction of the body. So it is important to understand how the combined treatment affects the specific immune response to the bacterial infection [1].

The lack of progress in development of antibiotics highlights a need for innovative approaches to deal with multidrug resistant infections. Iodine is uniquely effective in combating various infectious agents. Basically, all pathogenic bacteria are sensitive to iodine and no facts of acquired resistance against iodine-containing preparations have been recorded till now. Iodine possesses a universal potential in combating various infectious diseases. Despite high toxicity of the molecular iodine, its parenteral administration is possible in the form of complexes bearing iodine molecules in an active form. This creates a ground for further research on the use of iodine-containing preparations in medical practice as safe and effective means of treating and preventing infectious diseases.

Several complexes embracing iodine molecules in their organic moieties were synthesized in the Scientific Centre for Anti-Infectious Drugs (SCAID, Almaty, Kazakhstan). Our studies (unpublished) made us hypothesize that the iodine complexes could affect the immune cells. To examine this hypothesis, a complex of iodine with glycine (CC-195) has been created. A triiodide-Gly structure of a similar complex KS-25 was deposited in the CCDC database (https://www.ccdc.cam.ac.uk/) under an accession number 1036607.

An assessment of phagocytic and oxidative activities of monocytes and granulocytes affected by the combined therapy of cefazolin and the coordination compound СC-195 is presented here.

Bacterial pneumonia caused by the drug-resistant *S. aureus* SCAID OTT1-2022 was treated for five days. The therapy was carried out using three different treatment regiments: the iodine-containing coordination compound CC-195 in a dosage of 10.0 mg/kg; antibiotic cefazolin in a dosage of 25.0 mg/kg; and combination of CC-195 (10.0 mg/kg) with cefazolin (25.0 mg/kg). A group of intact mice was used as a negative control; whereas a group of infected mice left without any treatment served as a positive control. Blood samples were collected on the fifth day of treatment and five days after completion of treatment. This was done to evaluate how the immune system reacts to application of the drugs and discontinuation of the treatment.

Phagocytosis is the process of engulfing and destroying foreign particles, including bacteria. Monocytes and granulocytes are phagocytic cells and their ability to effectively engulf and destroy bacterial pathogens is crucial for protecting the body from infections [2,3].

After five days of treatment, there was a significant increase in monocytes in the mice treated solely either with CC-195 or with cefazolin compared to the intact mice and the infected animals of the positive control. In the group treated solely with CC-195, an increase in granulocytes (*p* < 0.05) was also observed compared to the group of infected and untreated animals (Table 1).

**Table 1:** Phagocytic activity of monocytes and granulocytes on the fifth day of treatment.

	Phagocytic activity					
Animal groups	Amount of phagocytic monocytes, %	P1*	Р2⁎⁎	Amount of phagocytic granulocytes, %	P1	Р2
Intact	77,61±7,69	-	-	91,61±1,19	-	-
Infected, untreated	81,64±9,21	-	-	86,14±9,32	-	-
CC-195 10 mg	98,55±1,04	˂0,0001	0,001	97,98±2,32	-	0,006
Cefazolin 25 mg	95,41±3,04	0,0006	0,01	85,53±4,90	-	-
CC-195 10 mg + Cefazolin 25 mg	89,21±9,33		-	90,15±2,79	-	-

⁎ Р1 – significance in comparison with intact animals.

⁎⁎ Р2 – significance in comparison with infected animals.

On the tenth day of observation (fifth days after the completion of therapy), a significant decrease of 29% in the number of monocytes and 12% in the number of granulocytes was observed in the group treated solely with cefazolin, compared to the infected untreated animals (Table 2).

**Table 2:** Phagocytic activity of monocytes and granulocytes on the fifth day after treatment completion.

	Phagocytic activity					
Animal groups	Amount of phagocytic monocytes, %	P1*	Р2⁎⁎	Amount of phagocytic granulocytes, %	P1	Р2
Intact	77,61±7,69	-	-	91,61±1,19	-	-
Infected, untreated	75,79±9,47	-	-	89,66±3,26	-	-
CC-195 10 mg	70,09±8,61	-	-	89,22±6,24	-	-
Cefazolin 25 mg	54,42±7,10	˂0,0001	˂0,0001	78,96±6,71	0,002	0,01
CC-195 10 mg + Cefazolin 25 mg	62,70±7,65	0,007	-	98,05±12,03	-	-

⁎ Р1 – significance in comparison with intact animals.

⁎⁎ Р2 – significance in comparison with infected animals.

Oxidative activity is another important aspect of the immune response to bacterial infections. Monocytes and granulocytes produce reactive oxygen species (ROS) as their normal function of killing bacteria [4].

Additionally, the number of cells producing reactive oxygen metabolites was determined. It has been shown that CC-195 monotherapy significantly increases (75 %) the number of monocytes, while the number of granulocytes has increased insignificantly at this condition (4.7 %) compared to the infected untreated animals (Table 3).

**Table 3:** Oxidative activity of monocytes and granulocytes the fifth day of treatment.

	Oxidizing activity					
Animal groups	Amount of oxygen-producing monocytes, %	P1*	Р2⁎⁎	Amount of oxygen-producing granulocytes, %	P1	Р2
Intact	55,89±14,39	-	-	98,34±0,82	-	-
Infected, untreated	44,69±12,07	-	-	91,91±4,58	0,007	-
CC-195 10 mg	78,29±2,43	0,003	˂0,0001	96,42±0,70	0,001	0,04
Cefazolin 25 mg	65,51±5,47	-	0,003	94,61±3,26	0,02	-
CC-195 10 mg + Cefazolin 25 mg	67,54±19,35	-	0,03	78,83±16,85	0,01	-

⁎ Р1 – significance in comparison with intact animals.

⁎⁎ Р2 – significance in comparison with infected animals.

On the tenth day of observation (five days after completion of the therapy), an interesting observation was that the amount of cells producing reactive oxygen metabolites changed differentially in the different groups of animals. In the animals treated solely with CC-195, the number of oxygen-producing monocytes has increased significantly: 54% higher than that in the healthy negative control animals, and 31.5% higher than in the infected and untreated positive control animals. In contrast to that, treatment of the infected animals solely with cefazolin caused a decrease in oxygen-producing monocytes (56.5%) than in the infected and untreated positive control animals that may indicate an immune suppression (Table 4).

**Table 4:** Oxidative activity of monocytes and granulocytes on the fifth day after treatment completion.

	Oxidizing activity					
Animal groups	Amount of oxygen-producing monocytes, %	P1*	Р2⁎⁎	Amount of oxygen-producing granulocytes, %	P1	Р2
Intact	55,89±14,39	-	-	98,34±0,82	-	-
Infected, untreated	65,43±18,48	-	-	95,8±3,36	-	-
CC-195 10 mg	86,05±12,93	0,009	0,04	98,81±0,86	-	-
Cefazolin 25 mg	28,39±3,78	0,001	0,0007	96,98±3,44	-	-
CC-195 10 mg + Cefazolin 25 mg	58,44±6,58	-	-	98,56±1,47	-	-

⁎ Р1 – significance in comparison with intact animals.

⁎⁎ Р2 – significance in comparison with infected animals.

All raw data from cytometry and summarized figures can be found in the Mendeley Data Repository. (http://dx.doi.org/10.17632/99hr4fn894.2).

## 3 Experimental Design, Materials and Methods

### 3.1 Sample collection

White outbred laboratory mice (both female and male, 4 weeks old, purchased from RSE at the PHV “Scientific and Practical Center for Sanitary and Epidemiological Expertise and Monitoringthe PHV” KZPP MNE RK, Almaty, Kazakhstan) were used. The mice were acclimatized to the conditions in the animal facility for one week before the beginning of the experiments. All mice were fed a standard rodent chow diet and kept under a 12-hour light and dark cycle. The animals were divided into five experimental groups (n=6 in each group): 1) Negative control – intact animals; 2) Positive control – infected and untreated animals; 3) Infected animals treated with 10.0 mg/kg of CC-195; 4) Infected animals treated with 25.0 mg/kg of cefazolin; 5) Infected animals treated with both: CC-195 and cefazolin as described above. The mice were infected with the clinical drug-resistant strain *S. aureus* SCAID OTT1-2022 (Accession no. CP102945). The drugs were administered once per day. During the combined therapy, CC-195 was applied 30 min in advance of the antibiotic. The therapy was carried out for five days. After five days of therapy and five days post-therapeutic period, in average 0.8 ml blood samples were collected into test tubes containing heparin as an anticoagulant. The heparinized blood samples were transferred to cytometer tubes.

### 3.2 Phagocytic and oxidative activity

The PHAGOTEST and BURSTTEST kits (BD Biosciences, USA) were used to determine phagocytic and oxidative activities of monocytes and granulocytes. All the procedures were carried out in accordance with the protocols of the manufacturer (BD Biosciences, USA). The samples were analysed on a FACSCalibur flow cytometer (BD Biosciences, United States) using the CellQuest Pro BD software using blue-green radiation (488 nm argon laser) [5]. The “live” gate was set up for these events which contain the same amount of DNA as diploid cells. Bacteria were excluded from the analysis by setting the fluorescence threshold through the FL2 and FL3 channels. Cells were collected in an amount of 10000-15000 leukocytes per sample.

### 3.3 Statistical data processing

For all data, the arithmetic means and standard deviations from the mean were calculated. The significance of differences between experimental data was assessed using GraphPad Prism version 6.00 for Windows (GraphPad Software, La Jolla California USA), using Two-way ANOVA. Values of the confidence level P > 0.05 were considered insignificant.

## CRediT authorship contribution statement

**Tamara A. Bukeyeva:** Writing – original draft, Methodology, Data curation. **Sabina G. Murzageldinova:** Funding acquisition, Investigation. **Aray A. Serikbay:** Investigation. **Arkadiy V. Krasnoshtanov:** . **Ilya S. Korotetskiy:** Conceptualization, Writing – review & editing, Project administration.

## Declaration of Competing Interests

The authors declare that they have no known competing financial interests or personal relationships that could have appeared to influence the work reported in this paper.

## Data Availability

Dataset phagocytic and oxidative activity after treatment by CC-195 and cefazolin (Original data) (Mendeley Data).
